# Polyamines induce adaptive responses in water deficit stressed cucumber roots

**DOI:** 10.1007/s10265-013-0585-z

**Published:** 2013-08-10

**Authors:** Jan Kubiś, Jolanta Floryszak-Wieczorek, Magdalena Arasimowicz-Jelonek

**Affiliations:** 1 Department of Plant Physiology, Poznań University of Life Sciences, Wołyńska 35, 60-637 Poznań, Poland; 2 Department of Plant Ecophysiology, Adam Mickiewicz University, Umultowska 89, 61-614 Poznań, Poland

**Keywords:** Cucumber, Lipoxygenase, Membrane damage, Polyamines, Proline, Water deficit

## Abstract

The aim of this study was to investigate the effect of exogenous polyamines (PAs) on the membrane status and proline level in roots of water stressed cucumber (*Cucumis sativus* cv. Dar) seedlings. It was found that water shortage resulted in an increase of membrane injury, lipoxygenase (LOX) activity, lipid peroxidation and proline concentration in cucumber roots during progressive dehydration. PA pretreatment resulted in a distinct reduction of the injury index, and this effect was reflected by a lower stress-evoked LOX activity increase and lipid peroxide levels at the end of the stress period. In contrast, PA-supplied stressed roots displayed a higher proline accumulation. The presented results suggest that exogenous PAs are able to alleviate water deficit-induced membrane permeability and diminish LOX activity. Observed changes were accompanied by an accumulation of proline, suggesting that the accumulation of this osmolyte might be another possible mode of action for PAs to attain higher membrane stability, and in this way mitigate water deficit effects in roots of cucumber seedlings.

## Introduction

Drought stress is a major limitation to crop productivity. Thus to develop crop plants with an enhanced tolerance to drought stress, a basic understanding of physiological, biochemical and gene regulatory networks is essential (Valliodan and Nguyen 2006). Plants also respond and adapt to water deficit at both cellular and molecular levels, for instance by the accumulation of osmolytes and proteins specifically involved in stress tolerance (Shinozaki and Yamaguchi-Shinozaki 2007). Abiotic stresses, especially water deficit, increase biosynthesis of both polyamines (PAs) (Bouchereau et al. 1999; Kubiś 2003, 2008; Yang et al. 2007) and compatible osmolytes, such as sugars, betaines and proline (Hare and Cress 1997).

Since a lack of water induces PA accumulation (Flores 1991; Flores and Galston 1984a, b; Kakkar and Sawhney 2002; Turner and Stewart 1986, 1988), a growing interest is observed in the possible involvement of PAs in the adaptive mechanism of plants to various environmental stresses (Bouchereau et al. 1999). Biological functions of PAs are attributed to their polycationic character at a physiological pH. Due to the presence of positively charged groups, PAs are able to bind strongly to negative charges in cellular components such as nucleic acids, proteins and phospholipids (Slocum et al. 1984; Smith 1985). An interaction of PAs with membrane phospholipids may stabilize membranes under stress conditions (Roberts et al. 1986), so its components may be buffered by PAs (Liu et al. 2007). PAs may directly or indirectly act as free radical scavengers (Bors et al. 1989). Spermine (Spm), which has four amino groups, is a more effective scavenger than spermidine (Spd), which has three amino groups, suggesting the involvement of amino groups in the inactivation of reactive oxygen species (ROS) (Besford et al. 1993). Indirectly, PAs are able to moderate the activities of scavenging system enzymes and alleviate oxidative stress intensity (Kubiś 2008). It has been shown that stress-tolerant plants increase endogenous PA levels to a greater extent than sensitive ones (Lee 1997). These molecules have been found to protect plants from abiotic stresses (Chattopadhayay et al. 2002; Liu et al. 2007; Shen et al. 2000); unfortunately, the precise mode of their action is not fully understood (Kakkar and Sawhney 2002). Additionally, through the enhancement of proline and betaine production PAs control and act as important osmoprotectant inducers in plat cells (Öztürk and Demir 2003).

A link between PAs and nitric oxide (NO) was shown by Tun et al. (2006) in *Arabidopsis thaliana* seedlings. These authors found that PAs induced NO biosynthesis, and another new mode of PA action via NO generation has already been confirmed that NO acts downstream of PAs in adaptive responses of cucumber leaves to water deficit stress (Yamasaki and Cohen 2006). Downregulation of NO by PAs was demonstrated in leaves of water-stressed cucumber plants by Arasimowicz-Jelonek et al. (2009). With regard to the finding reported by Gao et al. (2009), those higher levels of PAs and NO as well as the activities of arginine metabolism enzymes exist in roots rather than in leaves. It may be supposed that roots, especially new, fine ones, are much more exposed to many abiotic stresses, and are able to serve as an interface between plants and soil (Wells and Eissenstat 2003). According to the results of Gao et al. (2009), the authors also hypothesized that root-sourced molecules, i.e. PAs, are able to play a role in root-to-shoot signaling. This mode of action was proposed for PAs by Legocka and Kluk (2005). Results reported by those authors confirmed that osmotic and salt stresses induced PA biosynthesis in lupine roots, but Put accumulation in shoots, indicating root-to-shoot translocation, suggesting a potential role of PAs in root-to-shoot signaling.

Proline overproduction plays a highly protective role in plants that are exposed to abiotic stresses, conferring osmotic adjustment together with an increase in the levels of other osmolytes (Valliodan and Nguyen 2006). According to Yoshiba et al. (1997), compatible solutes could also be associated with lipids and proteins and thus counteracted negative dehydration effects on the cell structure and enzyme functioning. Other published data suggested other functions of proline, e.g. detoxification of ROS, and an interaction with the hydrophobic residue of proteins. The key role of proline in the response to water deficit has been demonstrated in transgenic tobacco that overexpressed proline biosynthesis enzymes (Kavi Kishor et al. 1995; Roosens et al. 2002). In turn, suppression of proline synthesis in transgenic plants resulted in increased sensitivity to water deficit (De Ronde et al. 2000). It was reported that transgenic petunia plants that overexpressed proline synthesis enzyme (pyrroline-5-carboxylate synthetase) genes from *Arabidopsis* (*AtP5CS*) and rice (*OsP5CS*) could withstand drought conditions longer than wild-type plants (Yamada et al. 2005).

In this study the role of PAs was analyzed in water deficit stress-induced changes in cucumber roots. Therefore it was attempted to determine whether exogenous PAs—Put, Spd and Spm, might modify membrane stability, lipoxygenase (LOX) activity and osmolyte-proline accumulation in cucumber seedling roots.

## Materials and methods

### Plant materials

Roots of cucumber seedlings (*Cucumis sativus* cv. Dar) were used as plant materials for experiments. After germination of sterilized seeds for 2 days at 24 °C seedlings were placed (5 per 1.0 dm^3^ beaker) and allowed to grow in a continuously aerated Hoagland’s solution in a growth chamber with a photoperiod of 16 h [light—250 μmol m^−2^ s^−1^ photosynthetically active radiation (PAR)] at the temperature of 24 °C (day) and 20 °C (night), and 60–70 % humidity. The nutrient solution was renewed once a week.

### PA treatment

One-month-old seedlings were taken out and divided into four groups. Their roots were immersed in the following, continuously aerated solutions: 1 mM K-phosphate buffer, pH 5.8 (control), or in buffer solutions containing additionally 1 mM Put, or Spd, or Spm, and maintained for 24 h under controlled conditions (22 °C, humidity 65 %, continuous light of 150 μmol m^−2^ s^−1^ PAR).

### Stress conditions

Half of the plants from each group was transferred into empty beakers and subjected to dehydration for 10 h (stressed plants). The other half of each plant group was maintained with their roots in 1 mM K-phosphate continuously aerated buffer, pH 5.8 (control plants). Then beakers with seedlings were placed in a growth chamber under controlled conditions (22 °C, humidity of 65 % at continuous light of 150 μmol m^−2^ s^−1^ PAR). Finally the roots were collected at 0, 5, and 10 h after withdrawal of water, and 24 h after rewatering (roots immersed in buffer) of 10 h-long stressed plants. Each sample contained 10 seedlings.

### Relative water content (RWC)

Indicating the level of water stress in roots, RWC was estimated according to Weatherley (1950), and calculated according to the following formula: RWC = [(fresh weight − dry weight)/(fresh weight at full turgor−dry weight)] · 100 %.

### Injury index

Electrolyte leakage from the roots was determined by the conductivity method and used as a criterion of injury. The amounts of electrolytes released from stressed or control tissues were compared to total electrolyte amounts released after boiling. The injury index was calculated according to a formula given by Flint et al. (1967): I_D_ = (L_D_ − L_0_)/(100 − L_0_) · 100 %, where I_D_ is the injury index, L_0_ is an electrolyte leakage from the control tissue in percent of the total electrolyte content, and L_D_ is an electrolyte leakage from the desiccated tissue in percent of the total electrolyte content. Determinations were performed in five replicates, each using the whole root system of an individual plant.

### LOX activity

Enzyme activity was measured according to Borrell et al. (1997). The increase in absorbance was monitored at 234 nm wavelengths. An absorbance increase of 0.001 was taken as one unit of LOX activity. Protein content in the extracts was determined according to Bradford (1976). Determinations were performed in five replicates and LOX activity was expressed in units per mg protein content.

### Proline determination

This metabolite content was performed according to the method given by Bates et al. (1973). The quantity of the colored reaction product of proline with ninhydric acid was measured. Absorbance was recorded at 520 nm and the amount of proline was calculated from the standard curve and expressed in μg × g^−1^ dry matter. Determination was performed in 5 replications.

### Histochemical detection of lipid peroxidation

For lipid peroxidation freshly harvested roots were stained in Schiff’s reagent for 60 min until pink color appeared, and then extra stain was removed by rinsing in potassium sulphite solution [0.5 % (w/v) K_2_S_2_O_5_ in 0.05 M HCl] as in Pompella et al. (1987). The lipid peroxidation range was estimated as thiobarbituric acid reactive substances in nmol per 1 g dry weight (DW).

### Statistical analysis

Analyses were performed in three to six replications and the data are presented as a mean ± standard deviation (SD). Experimental data were subjected to a one-way analysis of variance (ANOVA) and significant differences between means were determined by Tukey’s multiple range test. Data (stressed plants) significantly different from respective control (-PAs) at *P* < 0.05 were marked with a single asterisk on the figures.

## Results

**Fig. 1:**
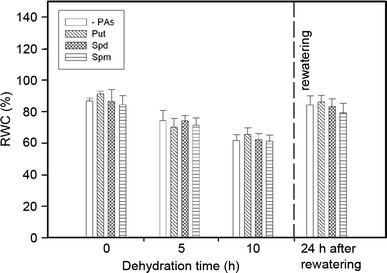
The effect of exogenous PAs on the relative water content during progressive dehydration of cucumber roots. Measurements were made 0, 5 and 10 h after water withholding and 24 h after rewatering. Prior to stress plants were immersed in: buffer (-PAs), 1.0 mM Put, 1.0 mM Spd and 1.0 mM Spm. Values indicate mean ± SE with *n* = 5

Water deficit greatly lowered RWC of cucumber roots, by as much as 60–65 % (Fig. 1) at the end of the 10-h stress period. In stressed plants treated with PAs, the dynamics of water content decrease was similar to that observed for untreated plants. There were no differences in RWC between PA-treated and untreated control plants. After rewatering the water content returned fast to the level recorded in unstressed plants (79–86 %).

**Fig. 2:**
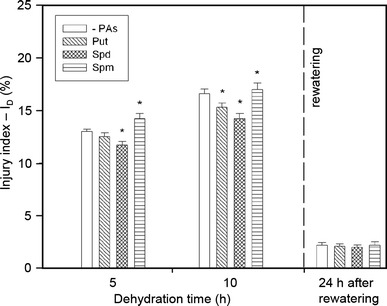
The effect of exogenous PAs on the injury index (I_D_) during progressive dehydration of cucumber roots. Measurements were made 5 and 10 h after water withholding and 24 h after rewatering. Prior to stress plants were immersed in: buffer (-PAs), 1.0 mM Put, 1.0 mM Spd and 1.0 mM Spm. Values indicate mean ± SE with *n* = 5. Data (stressed plants) significantly different from respective control: *P < 0.05

Water stress induced a marked increase in membrane permeability (Fig. 2), from approximately 12 % at 5 h to 17 % at 10 h. Generally, plants treated with PAs showed a significant reduction of stress-induced electrolyte leakage, depending on water stress duration. Pretreatment with Put and Spd resulted in a reduction of leakage consecutively by ca. 6 and 9 % at 5 h, respectively, and 7 and 20 % at 10 h, respectively, of dehydration in comparison with the untreated plants. In contrast, when seedlings were treated with Spm, a very slight increase in membrane damage was observed as compared to untreated seedlings. After rewatering, the injury index slowly decreased to the level comparable with that in the unstressed plants. In well-watered plants, either treated or untreated with PAs, practically no differences were observed in membrane permeability after 24 h of the experiment.

**Fig. 3:**
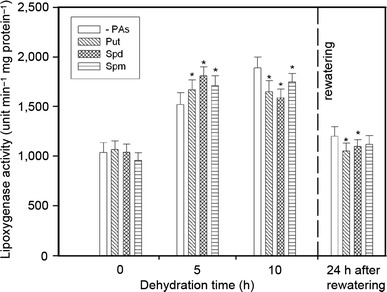
The effect of exogenous PAs on the LOX specific activity during progressive dehydration of cucumber roots. Measurements were made 0, 5 and 10 h after water withholding and 24 h after rewatering. Prior to stress plants were immersed in: buffer (-PAs), 1.0 mM Put, 1.0 mM Spd and 1.0 mM Spm. Values indicate mean ± SE with *n* = 6. *Asterisk* see Fig. 2

Water stress induced a significant increase in LOX activity (Fig. 3), which was noted as early as 5 h after dehydration (50 %). After 10 h of stress duration, an 80 % increase of LOX activity was recorded as compared to LOX of unstressed roots. PA application before water loss caused time-dependent changes in LOX activity: when compared with untreated roots, LOX activity in seedlings pretreated with Spd, Put and Spm increased by 20, 10 and 10 %, respectively, after 5 h of dehydration, then decreased by 10, 15 and 7 %, respectively, after 10 h of dehydration. After rewatering, LOX activity gradually dropped to the level observed in the unstressed plants. In seedlings pretreated with PAs, LOX activity remained slightly lower than in untreated ones.

**Fig. 4:**
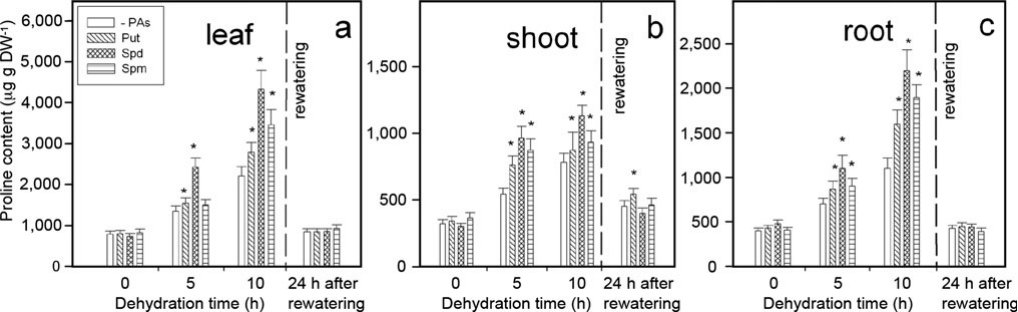
The effect of exogenous PAs on the proline content in leaf (**a**), shoot (**b**) and root (**c**) during progressive dehydration of cucumber seedlings. Measurements were made 0 to 10 h after water withholding and 24 h after rewatering. Prior to stress plants were immersed in: buffer (-PAs), 1.0 mM Put, 1.0 mM Spd and 1.0 mM Spm. Values indicate mean ± SE with *n* = 3. *Asterisk* see Fig. 2

Plant dehydration induced a gradual proline accumulation in cucumber seedlings (Fig. 4); over fivefold in leaves, four-fold in roots but only threefold in shoots, compare with plants not subjected to stress (time 0). When seedlings were treated with exogenously supplied PAs, a definitely higher accumulation of proline was observed after stress treatment: stressed roots pretreated with Put, Spd and Spm exhibited 24, 57 and 29 % higher accumulation, respectively, after 5 h; and 45, 100 and 72 % higher accumulation, respectively, after 10 h of stress duration, compared to PA-untreated stressed roots. Similarly, stressed shoots pretreated with Put, Spd and Spm exhibited 41, 78 and 59 % higher accumulation of proline after 5 h; and 16, 45 and 19 % higher accumulation after 10 h of stress duration, compared to PA-untreated shoots. Leaves pretreated with Put, Spd and Spm exhibited 15, 80 and 12 % increases, respectively, after 5 h; and 26, 96 and 57 % increases after 10 h of stress duration, compared to PA-untreated leaves. With regard to seedlings not subjected to water deficit stress and treated with PAs, all estimated parameters generally remained on a stable level during the experiment and these data were not presented in the figures.

**Fig. 5:**
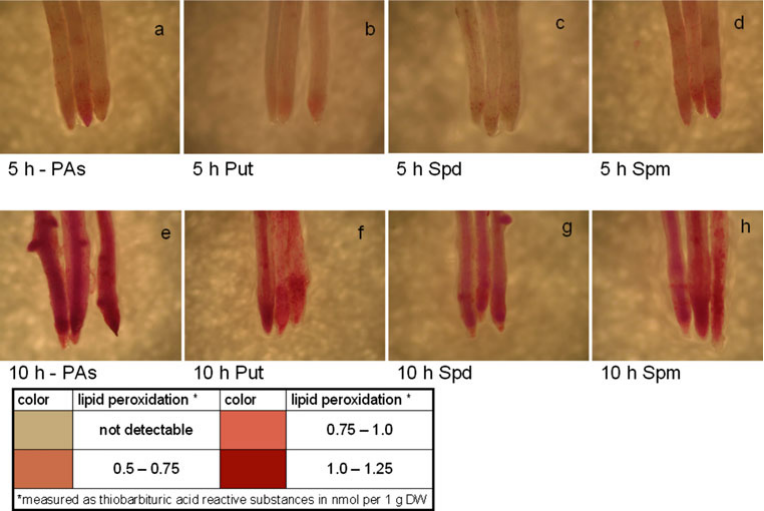
Histochemical detection of lipid peroxidation were performed by staining of Schiff’s reagent at 5 (**a**–**d**) and 10 h (**e**–**h**) of dehydration. The intensive *grey* (originally *pink* in a PDF version) color indicate a specific reaction for lipid peroxidation. Prior to stress plants roots were immersed in: buffer (-PAs **a** and **e**), 1.0 mM Put (**b** and **f**), 1.0 mM Spd (**c** and **g**) and 1.0 mM Spm (**d** and **h**)

For visualization of the membrane damage of root tissues, histochemical detection of lipid peroxidation was performed at 5 and 10 h of dehydration (Fig. 5). The intensive pink staining of Schiff’s reagent, a specific reaction for lipid peroxidation, was observed in PA-untreated root seedlings after 10 h of dehydration. In contrast, cucumber roots treated with PAs, especially with Spd, showed weaker dye (pink) staining compared to non-treated plants at 10 h of dehydration. At 5 h of dehydration, the pink coloring was mainly confined to the subapical zone of roots from untreated seedlings. In plants not subjected to dehydration or after rewatering, no pink staining was observed (data not shown). On the base of visualization no lipid peroxidation was detected and additionally the results of very small membrane damage (Fig. 2) were confirmed.

## Discussion

Plants respond in order to survive under water-deficit conditions via a series of physiological, cellular, and molecular processes culminating in stress tolerance. These adaptive changes include ABA synthesis and ABA-mediating phenomena, i.e. stomatal closure, PA and proline accumulation and changes within cell membranes (Lafitte et al. 2007; Shinozaki and Yamaguchi-Shinozaki 2007).

Obtained results provide evidence that cucumber seedlings treated with Spd exhibited a higher membrane stability under stress conditions, as reflected in a significant reduction of the stress-induced electrolyte leakage (Fig. 2) confirmed by visualization of the membrane damage (Fig. 5). Similar effects of higher levels of PA, Spd and Spm on the leakage of electrolytes and amino acids from salt-stressed rice roots have been reported by Chattopadhayay et al. (2002). They concluded that exogenous Spd and Spm are effective in triggering protection against cellular and macromolecular damage of rice plants during salinity stress, probably by maintaining membrane integrity and/or inhibiting protease and RNase activity during stress. On the other hand, it has been reported that PAs might mediate a decrease in ion fluxes across the vacuolar membrane by blocking fast-activating vacuolar channels under salt stress, as suggested by Bruggemann et al. (1998).

Progressive water shortage resulted in a water deficit-evoked increase in LOX activity (Fig. 3). Stress-induced LOX activation was also reported in desiccated soybean leaves (Kacperska and Kubacka-Zębalska 1989), in senescing muskmelon tissues (Lester 2000), and in barley and cucumber leaves subjected to water deficit (Arasimowicz-Jelonek et al. 2009; Kubiś 2006). In contrast, in chives tolerant to drought, a lower level of LOX activity under stress was noted (Egert and Tevini 2002).

Cucumber plants pretreated with PAs exhibited time-dependent changes in the stress-evoked activity of LOX as compared to PA-untreated stressed seedlings (Fig. 3). Barley plants pretreated with Spd prior to water shortage showed a higher, water deficit stress-evoked activity of LOX than PA-untreated stressed plants (Kubiś 2006). From our experiment we concluded that Spd protected soluble proteins against the water stress-induced decrease. Therefore, it may be speculated that an early increase in LOX activity (5 h) in the PA-pretreated water stressed tissues could be possibly due to LOX-protein protection. It has been shown that in maize Spd may bind to the 18-kD membrane protein (Tassoni et al. 2002) and in this way modulate activities of many enzymes such as protein kinases, phosphatases and ATPases (Tassoni et al. 1998). From the results cited above we may speculate that PA protection of LOX-proteins can also be possible. Moreover, lipid hydroperoxides produced by LOX served as substrates for stress-induced jasmonic acid (JA) biosynthesis (Rosahal 1996; Creelman and Mullet 1997) without modifying membrane stability.

Performed histochemical detection of lipid peroxidation resulted in intensive staining of Schiff’s reagent (originally pink) after 10 h of dehydration (Fig. 5, 10 h -PAs) and weaker staining after 5 h of dehydration (Fig. 5, 5 h -PAs) in whole regions of PA-untreated seedlings. In contrast, treatments of Spd and Put diminished staining, which was relatively confined to the tip regions of treated roots after 5 h (Fig. 5, 5 h Put and 5 h Spd) and less intensely in the whole root of treated roots after 10 h (Fig. 5, 10 h Put and 10 h Spd) of dehydration, suggesting membrane damages. In water-stressed barley leaves, Kubiś (2006) indicated that a significant twofold increase in LOX activity did not correspond with the relatively lower malonyldialdehyde (MDA) increase (48 %) after 24 h of dehydration. In addition, Spd pretreatment caused a three-fold increase in LOX activity, but only an approximately 30 % increase in MDA concentrations. It was suggested that the consumption of hydroperoxides produced by LOX can be a reason for lower membrane dysfunction, and PAs are engaged in alleviation of this effect in water-stressed barley seedlings. The involvement of methyl jasmonate in polyamine metabolism and in cell protection against another type of stress, i.e. pathogen infection, was earlier indicated (Biondi et al. 2001; Walters et al. 2002).

Our observations provide evidence that water deficit altered time-dependent proline accumulation in cucumber seedlings. The most significant, five-fold increase was observed in leaves, fourfold in roots and threefold in shoots. Bandurska and Stroiński (2003) also reported that in barley an earlier and significant increase was observed in leaves whereas in roots the accumulation was less significant. Observed in this work an effect of proline accumulation due to water deficit is in line with the results obtained by Handa et al. (1986), Raggi (1994), Bandurska (2000, 2001), and Knipp and Honermeir (2006), Najaphy et al. (2010). This amino acid is supposed to play a significant role in osmotic adjustment with regard to a reduction of osmotic potential due to the accumulation of solutes, and enables cells to maintain turgor during water deficit stresses (Ashraf and Foolad 2007; Lopez-Carrion et al. 2008). Our data indicated that cucumber seedlings, treated with PAs, especially with Spd, prior to water deficit, exhibited a definitely higher stress-evoked proline accumulation. In turn, the induction of proline accumulation might be an important mechanism for plants to tolerate severe stress conditions (Ruan et al. 2004).

It is well documented that PAs are able to induce adaptive changes in water stressed plants; it is of the prime importance to maintain plasma membrane integrity under water deficit conditions. Pretreatment of cucumber roots with PAs resulted in a distinct reduction of the injury index and this observed PA effect was mirrored by a lower stress-evoked LOX activity increase in stressed seedlings at a severe water deficit. The other possible mechanism of the PA mode of action is an osmotic adjustment due to the accumulation of solutes, enabling cells to maintain turgor during water shortage. In seedlings supplied with PAs prior to stress, a significantly higher osmolyte-proline accumulation was recorded.

It allows us to speculate that under water deficit stress accumulated PAs could act as signal molecules and may trigger efficient adaptive mechanisms, resulting in the alleviation of negative drought effects. Additionally, these root-sourced signaling molecules are able to play an important role in root-to-shoot signaling and help to adapt plants to drought and prevent crops from serious stress-caused damages.

## Abbreviations

LOX: Lipoxygenase
PAs: Polyamines
Put: Putrescine
Spd: Spermidine
Spm: Spermine
